# Unlocking the Potential of AI in EUS and ERCP: A Narrative Review for Pancreaticobiliary Disease

**DOI:** 10.3390/cancers17071132

**Published:** 2025-03-28

**Authors:** Catarina Cardoso Araújo, Joana Frias, Francisco Mendes, Miguel Martins, Joana Mota, Maria João Almeida, Tiago Ribeiro, Guilherme Macedo, Miguel Mascarenhas

**Affiliations:** 1 Precision Medicine Unit, Department of Gastroenterology, São João University Hospital, Alameda Professor Hernâni Monteiro, 4200-427 Porto, Portugal; anacatarina.araujo98@gmail.com (C.C.A.); joanafriasdasilva@gmail.com (J.F.); francisco.cnm@gmail.com (F.M.); miguelpedro96@gmail.com (M.M.); joanamfmota8@gmail.com (J.M.); maj.almeida.14@gmail.com (M.J.A.); tiagofcribeiro@outlook.com (T.R.); guilhermemacedo59@gmail.com (G.M.); 2 WGO Gastroenterology and Hepatology Training Center, Alameda Professor Hernâni Monteiro, 4200-427 Porto, Portugal; 3 Faculty of Medicine, University of Porto, Alameda Professor Hernâni Monteiro, 4200-427 Porto, Portugal

**Keywords:** artificial intelligence, endoscopic ultrasound, endoscopic retrograde cholangiopancreatography, cholangioscopy, cystic pancreatic lesions, solid pancreatic lesions, indeterminate biliary strictures, deep learning

## Abstract

Artificial Intelligence (AI) is transforming pancreaticobiliary endoscopy by enhancing diagnostic accuracy, procedural efficiency, and clinical outcomes. This narrative review explores AI’s applications in endoscopic ultrasound (EUS) and endoscopic retrograde cholangiopancreatography (ERCP), emphasizing its potential to address diagnostic and therapeutic challenges in pancreaticobiliary diseases. In EUS, AI improves pancreatic mass differentiation, malignancy prediction, and landmark recognition, demonstrating high diagnostic accuracy and outperforming traditional guidelines. In ERCP, AI facilitates precise biliary stricture identification, optimizes procedural techniques, and supports decision-making through real-time data integration, improving ampulla recognition and predicting cannulation difficulty. Additionally, predictive analytics help mitigate complications like post-ERCP pancreatitis. The future of AI in pancreaticobiliary endoscopy lies in multimodal data fusion, integrating imaging, genomic, and molecular data to enable personalized medicine. However, challenges such as data quality, external validation, clinician training, and ethical concerns—like data privacy and algorithmic bias—must be addressed to ensure safe implementation. By overcoming these challenges, AI has the potential to redefine pancreaticobiliary healthcare, improving diagnostic accuracy, therapeutic outcomes, and personalized care.

## Simple Summary

Artificial Intelligence (AI) is rapidly transforming pancreatic and bile duct procedures. This review explores how AI is being used to improve patient care in these areas. By focusing on two major procedures—endoscopic ultrasound and endoscopic retrograde cholangiopancreatography—this review highlights how AI enhances accuracy, streamlines procedures, and minimizes complications. The authors discuss how AI can help identify conditions like mucinous cystic pancreatic lesions, pancreatic ductal adenocarcinoma, or malignant biliary strictures more reliably than current methods and even predict potential difficulties during procedures. Looking ahead, AI has the potential to integrate genetic and molecular information, paving the way for more personalized treatments. However, this review also emphasizes the need to address challenges like ensuring high-quality data, training healthcare professionals, and resolving ethical issues. This work aims to guide the medical community toward safely implementing AI to revolutionize care for pancreatic and bile duct diseases.

## 1. Introduction

Artificial Intelligence (AI) is a term used to describe the ability of technology to simulate human intelligence. Nowadays, we are witnessing intense research focused on AI applications in the medical field, which can offer us unprecedented opportunities for diagnosis and treatment, thereby improving the quality of healthcare in clinical practices.

A key component of AI is Machine Learning (ML), which refers to a system’s ability to learn from data and improve its performance over time. ML includes various approaches, such as supervised learning, where models are trained on labeled data, and unsupervised learning, where patterns and structures are identified without explicit labels [1,2]. It is a subtype of AI that relies more heavily on human intervention to learn. Deep Learning (DL) is a subset of ML that relies on artificial neural networks (ANNs), which allow for multiple layers of features to be extracted from unprocessed data to create more complex predictive outputs with a reduced need for human guidance. Neural networks, in turn, are a collection of algorithms designed to process data and generate an output with minimal errors, mimicking the synapses of the human brain [3].

Pancreaticobiliary diseases present significant diagnostic and therapeutic challenges due to their complex anatomical location, the variety of diseases involved, and overlapping symptoms, particularly between benign and malignant conditions. Accurate and timely diagnosis is critical, as treatment strategies vary considerably based on disease type. Pancreaticobiliary endoscopy includes techniques such as endoscopic ultrasound (EUS), endoscopic retrograde cholangiopancreatography (ERCP), and cholangioscopy, but these tools do have limitations. For instance, EUS has a long learning curve and is highly operator-dependent, making it a technique where small lesions may be overlooked [3]; ERCP, on the other hand, can be technically challenging due to the difficulty in cannulating the bile duct [4]; and cholangioscopy is challenged with extrinsic biliary strictures and distal biliary lesions, the lack of standardized classifications to distinguish between malignant and benign lesions, and the low accuracy and sensitivity of digital single-operator cholangioscopy (DSOC)-guided biopsies associated with a low sampling rate, since only a small amount of tissue can be obtained [5,6].

But how exactly has AI contributed to the field of pancreaticobiliary diseases? Through sophisticated algorithms and data analysis, AI is enhancing the accuracy, efficiency, and reproducibility of pancreaticobiliary endoscopy. AI-powered tools, such as Computer-Assisted Detection (CADe) and Computer-Assisted Diagnosis (CADx) systems, assist in lesion detection and differentiation in real time during endoscopic procedures, which might help identify suspicious areas that may require biopsy, microscopic examination, or further clinical evaluation [7]. Furthermore, AI applications extend beyond diagnosis to include predictive analytics and real-time procedural guidance, demonstrating their versatility and potential to revolutionize pancreaticobiliary healthcare.

Despite these advancements, significant hurdles remain, including the need for robust datasets, generalizable models, and broader clinical validation. This review explores the current applications of AI in pancreaticobiliary endoscopy, emphasizing its role in enhancing ERCP and EUS, and highlights future opportunities for integrating AI into clinical practice.

## 2. Applications of AI in Diagnostic Pancreaticobiliary Endoscopy

### 2.1. Endoscopic Ultrasound

**Figure 1:**
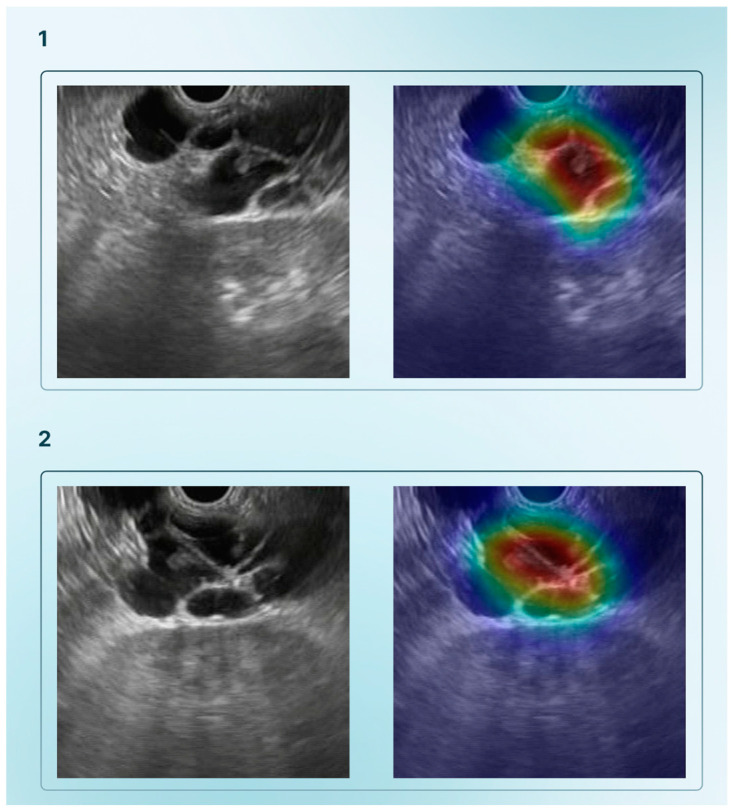
Heatmap analysis showing the prediction of the algorithm for the identification of two different mucinous pancreatic cystic lesions (**1**,**2**).

Concerning EUS, Table 1 summarizes the main studies to date on the applicability of AI for diagnostic use. In fact, AI has been instrumental in the differentiation of pancreatic masses, including cystic and solid lesions. Pancreatic cystic lesions (PCLs) pose a major challenge due to their significant prevalence and different malignant potential [8]. Indeed, there are several types of PCLs, such as mucinous cystic neoplasms (MCNs), including intraductal papillary mucinous neoplasms (IPMNs), serous cystic neoplasms (SCNs), and pancreatic pseudocysts (PPCs). It is known that malignancy occurs virtually only in patients with mucinous-phenotype PCLs. Vilas-Boas et al. explored this topic, developing a DL algorithm to distinguish mucinous from non-mucinous pancreatic cysts based on EUS images, achieving remarkable results with an accuracy of 98.5%, a sensitivity of 98.3%, and a specificity of 98.9% [9]. Figure 1 illustrates this application by the creation of heatmaps displaying the algorithm’s prediction for identifying mucinous pancreatic cystic lesions. Similarly, Nguon et al. developed a DL model to differentiate pancreatic MCNs from SCNs using EUS. The model achieved up to 82.75% accuracy and an area under the curve (AUC) of 0.88 [10].

Another upcoming application of AI consists of the differentiation between pancreatic solid lesions, such as pancreatic ductal adenocarcinomas (PDACs) and pancreatic neuroendocrine tumors (PNETs), and other lesions, such as autoimmune pancreatitis (AIP) and chronic pancreatitis (CP). This distinction is crucial given the poor prognosis associated with PDACs—it often presents at an advanced stage with a 5-year survival rate of less than 10% [11]. Several EUS complementary techniques are used for differential diagnosis, such as grayscale, color doppler, contrast enhancement, and elastography [12]. However, diagnosis remains highly operator-dependent, and accurate cytopathological diagnosis of PDACs is challenging, especially for inexperienced pathologists [13].

By analyzing a large number of images in real time, AI’s capabilities are particularly advantageous. Marya et al. developed a convolutional neural network (CNN)-based model to differentiate AIP from PDAC, CP, and the normal pancreas. The model demonstrated high sensitivity and specificity across various comparisons, such as 90% and 93% for AIP versus PDAC and 90% and 85% for AIP versus all conditions combined, respectively [14]. Udriștoiu et al. expanded this approach by integrating a CNN with long short-term memory (LSTM) models to classify images into categories such as PNET, PDAC, or chronic pseudotumoral pancreatitis. Their study utilized advanced imaging techniques, including grayscale, color doppler, arterial and venous phase contrast enhancement, and elastography, achieving similarly high diagnostic performance [12].

More recently, Saraiva et al. developed a CNN not only to detect and distinguish pancreatic solid lesions, such as PDAC and PNET, but also to differentiate cystic lesions, such as mucinous and non-mucinous lesions, involving four international reference centers. The CNN had an accuracy of 99.1%, 99.0%, and 99.8% for identifying normal pancreatic tissue and mucinous and non-mucinous cystic neoplasms, respectively. The accuracy of the distinction between PDAC and PNET was 94.0% [15].

AI has also proved to have the potential to predict malignancy in patients with IPMNs. Kuwahara et al. developed an AI-based algorithm to evaluate malignancy risk in IPMNs using EUS images. This model not only achieved significant predictive success but also surpassed the diagnostic performance of human preoperative evaluation and conventional prognostic techniques, highlighting the transformative impact of AI in preoperative cancer risk stratification [16]. Within this context, Machicado et al. conducted a post hoc analysis of a single-center prospective study evaluating EUS-guided needle-based confocal laser endomicroscopy (EUS-nCLE), aiming to apply predictive computer-aided detection and diagnosis (CAD) and AI algorithms to enhance diagnostic accuracy and risk stratification of IPMNs. Their study encompassed 15,027 video frames from 35 patients with histopathologically confirmed IPMNs. For detecting high-grade dysplasia/adenocarcinoma in IPMNs, the diagnostic performance of the AI algorithms was compared to that of the American Gastroenterological Association and revised Fukuoka guidelines, achieving superior results with improved sensitivity and accuracy while maintaining comparable specificity [17].

In addition, AI can help simplify the learning and identification of anatomical landmarks during EUS, improving its training and quality control, as described in a paper published by Zhang et al. This group built a real-time automated system called BP MASTER, an EUS station recognition and pancreas segmentation system using DL, which served as a real-time transducer positioning and pancreas vision loss monitoring system. Thus, the potential of AI to shorten the learning curve of pancreatic EUS was demonstrated since the recognition accuracy of the trainee station increased from 67.2% to 78.4% (*p* > 0.01), with a classification and segmentation performance comparable to that of EUS experts [18]. The same group developed a DL-based system for real-time evaluation of the bile duct (BD) during linear EUS. This system enables precise BD segmentation, automatic diameter measurement, and station recognition, shortening physician workflows. Notably, the CNN-based system outperformed senior EUS endoscopists and demonstrated accuracy comparable to that of expert EUS practitioners [19].

AI has also enhanced the utility of elastography in EUS. Saftoiu et al. pioneered this application in 2008, using neural networks to analyze EUS elastography images based on hue histograms. Their study achieved a sensitivity of 91.4%, a specificity of 87.9%, and an accuracy of 89.7% for differentiating benign from malignant lesions [20]. In 2012, the same authors carried out a larger, multicenter study validating this approach [21].

EUS-guided fine needle aspiration and biopsy (EUS-FNA/B) remains the mainstay of preoperative pathological diagnosis. However, these techniques often face challenges related to low specimen volume with isolated cancer cells and high contamination of blood, inflammatory, and digestive tract cells. Thus, there is room for AI to improve this process. Naito et al. developed a DL model that analyzed EUS-FNB histopathological images to detect isolated cancer cells, achieving an AUC of 0.984, a sensitivity of 93.02%, and a specificity of 97.06% [22]. Similarly, Ishikawa et al. conducted a study to assess the usefulness of AI in predicting the diagnosable material for histology using fresh specimens. The aim was to develop an AI-based method that could be an alternative to macroscopic on-site evaluation (MOSE) for evaluating EUS-FNB specimens in pancreatic diseases. They concluded that the AI-based method using contrastive learning was comparable to expert-driven MOSE [23].

Further advancements were demonstrated by Qin et al., who introduced a hyperspectral imaging (HSI)-based CNN algorithm to enhance the diagnostic process for pancreatic EUS-FNA cytology specimens. Comparing an RGB-based CNN with an HSI-based CNN, they demonstrated the superior accuracy of the HSI model in distinguishing malignant from benign pancreatic cells. For the test set, the RGB model achieved 82.4% accuracy, while the HSI model reached 88.05%. By incorporating the SimSiam algorithm, the HSI model’s performance improved further, achieving 92.04% accuracy [13]. These findings underscore the potential of HSI to capture diagnostic information beyond the scope of conventional imaging methods.

Finally, the applicability of AI in contrast-enhanced EUS (CE-EUS) has also been studied. In fact, it is known that when CE-EUS is combined with EUS-FNA, the sensitivity of the latter increases since CE-EUS helps avoid sampling necrotic or inflammatory tissue, thereby increasing the diagnostic yield of EUS-FNA [24]. Tang et al. demonstrated this with their CH-EUS MASTER system, which integrates DL models for real-time pancreatic mass capture and segmentation (Model 1), a benign and malignant identification model (Model 2), and an EUS-FNA-targeted auxiliary system. Afterward, a single-center randomized-controlled trial (RCT) was conducted to evaluate this system. The accuracy, sensitivity, specificity, positive and negative predictive values (PPV and NPV), and AUC of CH-EUS MASTER were significantly better than those of the endoscopists [25].

**Table 1:** Overview of the published work on the application of AI in EUS in pancreatic disorders. AI, Artificial Intelligence; DL, Deep Learning; CNN, convolutional neural network; ANN, artificial neural network; SEN, sensitivity; SPE, specificity; AUC, area under the curve; EUS, endoscopic ultrasound; EUS-FNA, endoscopic ultrasound-guided fine needle aspiration; EUS-FNB, endoscopic ultrasound-guided fine needle biopsy; MLP, multilayer perceptron; LSTM, long short-term memory; CEH-EUS, contrast-enhanced harmonic endoscopic ultrasound; NP, normal pancreas; PDAC, pancreatic ductal adenocarcinoma; ADC, adenocarcinoma; CP, chronic pancreatitis; AIP, autoimmune pancreatitis; CPP, chronic pseudotumoral pancreatitis; PNET, pancreatic neuroendocrine tumor; MFP, mass-forming pancreatitis; PCL, pancreatic cystic lesion; PSL, pancreatic solid lesion; PCN, pancreatic cystic neoplasms; M-PCN, mucinous pancreatic cystic neoplasm; MCN, mucinous cystic neoplasm; NM-PCN, non-mucinous pancreatic cystic neoplasm; SCN, serous cystic neoplasm; IPMN, intraductal papillary mucinous neoplasm; ROI, region of interest; NK, not known.

Publication Author, Year	Study Aim	Centers, n	Exams, n	Total nr Frames	Lesions nr Frames	Types of CNN	Dataset Methods	Analysis Methods	Classification Categories	SEN	SPE	AUC
Săftoiu et al., 2008 [20]	Assess accuracy of real- time EUS elastography for detecting malignant pancreatic tumors using postprocessing software for analysis	2	NK	NK	NK	ANN (MLP)	A hue histogram was calculated for each frame, summarizing it into a single numerical form, and then averaged across frames for each patient	Train–test split, employing a 10-fold cross–validation	Normal pancreas, CP, pancreatic cancer, and PNET	91.4%	87.9%	0.932
Săftoiu et al., 2012 [21]	Assess accuracy of real-time EUS elastography in focal pancreatic lesions using computer-aided diagnosis by ANN analysis	13	774	96,750	NK	ANN (MLP)	Manually labeled and selected tumor regions in each frame for analysis	Train–test split, employing a 10-fold cross–validation	CP, pancreatic cancer	87.59%	82.94%	0.94
Kurita et al., 2019 [26]	Evaluate the use of AI and DL in analyzing cyst fluid to differentiate between malignant and benign PCLs, comparing it to tumor markers, amylase, and citology	1	NK	NK	NK	ANN	Frame labeling of all datasets (malignant cystic lesions were labeled as “1” and benign lesions as “0”)	Train–test split (80–20% with five-fold cross–validation)	Benign vs. malignant cystic pancreatic lesions	95.7%	91.9%	0.966
Kuwahara et al., 2019 [16]	Evaluate the use of AI via a DL algorithm to predict malignancy of IPMNs using EUS images	1	NK	3970 (with data augmentation 508,160)	NK	ResNet	Frame labeling of all datasets (malignant were labeled as “1” and benign lesions as “0”)	Train–test split (90–10% with 10-fold cross–validation)	Benign vs. malignant IPMN	95.7%	92.6%	0.98
Naito et al., 2021 [22]	Train a DL model to assess PDAC on EUS-FNB of the pancreas in histopathological whole-slide images	1	NK	532	267	EfcientNet-B12	Manual annotations (adenocarcinoma vs. non-adenocarcinoma)	Train–validation–test	ADC vs. non-ADC	93.02%	97.06%	0.984
Marya et al., 2021 [14]	Create an EUS-based CNN model trained to differentiate AIP from PDAC, CP, and NP in real time	1	NK	1,174,461	NK	ResNet	Video frames and still images were manually annotated and extracted from EUS (AIP, PDAC, CP, and NP)	Train–validation–test (60–20–20%)	PDAC, AIP, CP, or NP	90%	78%	NK
Udristoiu et al., 2021 [12]	Real-time diagnosis of focal pancreatic masses using a hybrid CNN-LSTM (long short-term memory) model on EUS images	NK	NK	1300 (with data augmentation 3360)	PDAC: 1240; CPP: 1120; PNET: 1000	Hybrid CNN-LSTM	Manual annotations (PDAC, CPP, or PNET)	Train–validation–test (80% of images were chosen randomly for validation or training and 20% for testing)	CPP, PNET, PDAC	98.60%	97.40%	0.98
Tonozuka et al., 2021 [27]	Detect PDAC from EUS images using a DL model	1	NK	1390 static images (with data augmetation 88,320)	NK	CNN and pseudocolored heatmap	Frame labelling of all datasets (PDAC, CP, or NP)	Train–validation–test (training–validation set ratio: 90–10%; 10-fold cross–validation)	PDAC, CP, NP	92.4%	84.1%	0.940
Nguon et al., 2021 [10]	Develop a CNN to differentiate between MCN and SCN	1	NK	211	MCN: 130; SCN: 81	ResNet	ROI around the cysts in EUS images were manually selected	Train–test (10 patients from each class—MCN and SCN—were used for testing, while the rest were used for training)	MCN, SCN	Single-ROI: 81.46%; Multi-ROI: 76.06%	Single-ROI: 84.36%; Multi-ROI: 84.55%	Single-ROI: 0.88; Multi-ROI: 0.84
Ishikawa et al., 2022 [23]	Develop a AI-based method for evaluating EUS-FNB specimens in pancreatic diseases	1	NK	298	NK	AlexNet for DL and SimCLR for contrastive learning	NK	Train–validation–test	PDAC, MFP, AIP, PNET, IPMNs, and metastatic pancreatic tumor	DL: 85.8%; Contrastive learning: 90.3%	DL: 55.2%; Contrastive learning: 53.5%	0.879
Vilas-Boas et al., 2022 [9]	Develop a DL algorithm that differentiates mucinous and non-mucinous pancrea	1	28	5505	Mucinous PCLs: 3725; Non-mucinous PCLs: 1780	Xception	Frame labeling of all datasets (Mucinous PCLs and non-mucinous PCLs)	Train–validation–test (80–20%)	Normal pancreatic parenchyma, mucinous PCLs, and non-mucinous PCLs	98.3%	98.9%	1
Qin et al., 2023 [13]	Develop a hyperspectral imaging-based CNN algorithm to aid in the diagnosis of pancreatic cytology specimens obtained by EUS-FNA/B	1	NK	1913	890	ResNet18+ SimSiam	NK	Train–validation–test (60–20–20%)	PDAC cytological specimens, benign pancreatic cells	93.10%	91.23%	0.9625
Tang et al., 2023 [25]	Develop a DL based system, for facilitating diagnosing pancreatic masses in CEH-EUS, and for guiding EUS-FNA in real-time, to improve the ability of distinguishing between malignant and benign pancreatic masses	1	NK	Model 1: 4342; Model 2: 296	Model 1: 3546; Model 2: 167 (PDAC)	Model 1: Unet++ (ResNet-50 used as a backbone)	Manual labeling of all data sets (benign vs. malignant lesions)	Train–test (80–20%) for both models	Benign vs. malignant lesions	- Identification benign/malign pancreatic masses: 92.3%; - Guiding EUS-FNA: 90.9%	- Identification benign/malign pancreatic masses: 92.3%; - Guiding EUS-FNA: 100%	- Identification benign/malign pancreatic masses: 0.923; - Guiding EUS-FNA: 0.955
Saraiva et al., 2024 [15]	Develop a CNN for detecting and distinguish PCN (namely M-PCN and NM-PCN) and PSL (PDAC and PNET)	4	378	126,000	M-PCN: 19,528; NM-PCN: 8175; PDAC: 64,286; PNET 29,153	ResNet	Each image had a predicted classification related to the highest probability	Train–test split (90–10%)	M-PCN; NM-PCN; PDAC; PNET; NP	M-PCN: 98.9% NM-PCN: 99.3% PDAC:98.7% PNET:83.7%	M-PCN: 99.1% NM-PCN: 99.9% PDAC: 83.7% PNET: 98.7%	NK

### 2.2. Endoscopic Retrograde Cholangiopancreatography and Cholangioscopy

AI has also been transformative in ERCP, particularly for diagnosing biliary strictures. Indeterminate biliary strictures (IDBSs) refer to strictures without an obvious mass on imaging and without definitive tissue diagnosis [28]. IDBSs account for 20% of biliary strictures after initial evaluations, including EUS and ERCP, which is related to the suboptimal sensitivity associated with traditional sampling techniques such as brush cytology and forceps biopsy (23–81%, according to a recent review [29]). Cholangioscopy, by enabling direct bile duct visualization, improves diagnostic accuracy, with sensitivity for malignant strictures reported at approximately 86.7% [30]. Visual indicators of malignancy include the presence of masses or tumors, irregular, ulcerated, infiltrative, or friable surfaces, neovascularization or dilated tortuous vessels, and papillary projections [31]. DSOC-guided biopsies do, however, exhibit inadequate sensitivity rates of roughly 74% [32]. Thus, making the etiological diagnosis of biliary strictures is often a challenging task because traditional exams have disappointing performance metrics. The importance of a correct diagnosis is especially crucial since the associated malignant conditions have a very poor prognosis, not only because of the low survival rates but also because of the high-risk surgeries that patients usually undergo [33]. In addition, there is sometimes a high consumption of resources and economic cost associated with repeat examinations [34]. These challenges highlight the need for improved diagnostic approaches to better manage these complex lesions. Therefore, the introduction of AI into ERCP and cholangioscopy seems promising. Table 2 compiles the main AI research studies in this field.

**Figure 2:**
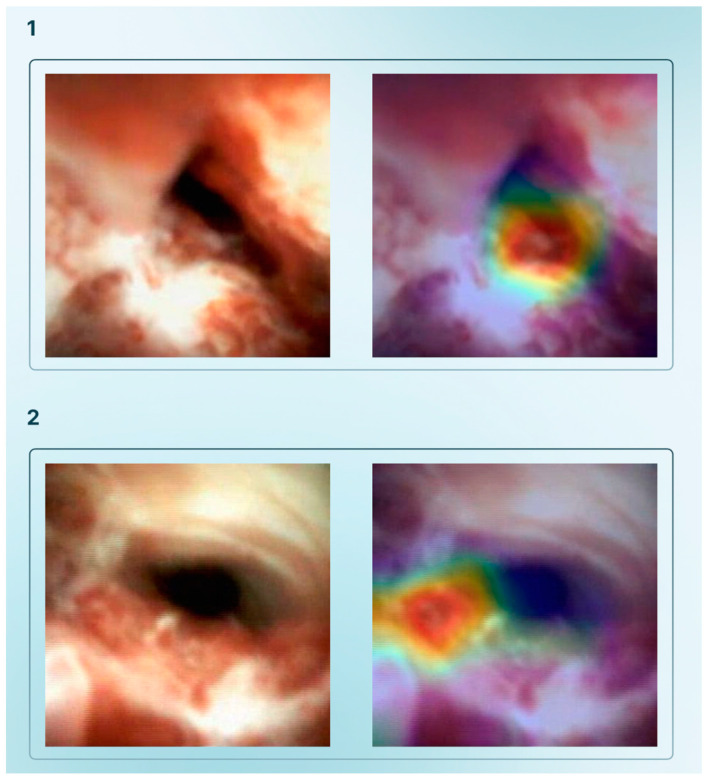
Heatmap analysis showing the prediction of the algorithm for the identification of two different malignant strictures (**1**,**2**).

In 2022, Saraiva et al. developed a DL algorithm that accurately detected and differentiated malignant strictures from benign biliary conditions, showing that the introduction of AI algorithms into DSOC systems may significantly increase its diagnostic yield [35]. In 2023, the same group conducted an international multicenter study, developing a new CNN model to differentiate the etiology of biliary strictures. The model successfully achieved an overall accuracy of 82.9%, with a sensitivity of 83.5% and a specificity of 82.4%, as well as an AUC of 0.92 [36]. Figure 2 shows two examples of the heatmap analysis that accurately predicted where malignant strictures were localized. Similarly, Marya et al. applied CNN technology to cholangioscopy images with the aim of classifying biliary strictures, achieving an accuracy of 90.6%, far surpassing traditional brush cytology and biopsy techniques (62.5%, *p* = 0.04; 60.9%, *p* = 0.03, respectively) [37].

Further advancements in cholangioscopy include Zhang et al.’s MBSDeiT system, which autonomously identifies qualified images for malignancy assessment and then predicts their malignancy in real time. This model achieved high accuracy in the automatic detection of qualified images, with an AUC of 0.963–0.973 across internal and external testing datasets. The system also showed strong results in identifying malignant biliary strictures, with an AUC of 0.971–0.99 across the same datasets. Finally, these findings were compared to those of both experienced and novice endoscopists, demonstrating the system’s superiority [38].

Lastly, in 2024, the first transatlantic multicenter study based on DSOC images from three high-volume reference centers was published by Saraiva et al. The study validated a CNN model on a large dataset of DSOC images, enabling the automatic detection of malignant strictures and their morphological characterization. The classification was compared to the gold standard of DSOC biopsies or surgical specimens. The results were excellent, showing a great discriminatory capability for IDBSs and confirming robust performance across diverse demographic contexts and various DSOC devices, effectively addressing interoperability challenges [39].

The first case series of an AI algorithm for the automatic classification of biliary strictures was recently published by the same group, highlighting the real-life application of a previously described DL algorithm in real time [36]. These case studies confirmed the CNN’s ability to operate effectively and provide predictions as suggested in earlier research, correctly predicting the malignant etiology of biliary strictures of two patients and a very low probability of malignancy of others. Furthermore, the study was conducted across three major centers using two different cholangioscopy systems, underlining its great power of generalization [40].

A final note should be made about probe-based confocal laser endomicroscopy (pCLE), an advanced technique that enables real-time, in vivo visualization of biliary strictures, allowing for the acquisition of real-time microscopic images of the biliary epithelium. This provides histological insights that otherwise would not be possible during ERCP [41]. Several published studies support the efficacy of this procedure in diagnosing IDBSs, with DSOC-guided pCLE reported to have a sensitivity of 93% and specificity of 82% for detecting neoplasia [42]. However, despite these promising results, this technology remains expensive, and the required equipment is not globally available. To the best of our knowledge, no studies have explored the application of AI in procedures utilizing pCLE. However, it is worth highlighting a study by Robles-Medranda et al., which compared the performance of a DSOC-based AI model with DSOC-guided pCLE for identifying malignant biliary strictures. Their retrospective study evaluated four diagnostic methods for biliary strictures in each patient: direct visualization with DSOC, DSOC-pCLE, offline DSOC-based AI model analysis (performed on DSOC recordings), and DSOC/pCLE-guided biopsies. The results demonstrated similar diagnostic performance across all methods; however, larger prospective studies are required to further validate these results [43].

**Table 2:** Overview of the published work on the application of AI in cholangioscopy in IDBSs. AI, Artificial Intelligence; DL, Deep Learning; CNN, convolutional neural network; SEN, sensitivity; SPE, specificity; AUC, area under the curve; DSOC, digital single-operator cholangioscopy; BS, biliary stricture; PP, papillary projection; TV, tumor vessel; NK, not known.

Publication Author, Year	Study Aim	Center n	Exams n	Total nr Frames	Lesion nr Frames	Types of CNN	Dataset Methods	Analysis Methods	Classification Categories	SEN	SPE	AUC
Ribeiro et al., 2021 [44]	Develop an AI algorithm for automatic detection of PP in DSOC images	1	NK	3920	1650	Xception	Frame labeling of all datasets (benign finding vs. PP)	Train–validation (80–20%)	Benign findings or PP	99.7%	97.1%	1
Saraiva et al., 2022 [35]	Develop a CNN-based system for automatic detection of malignant BSs in DSOC images	1	NK	11,855	9695	Xception	Frame labeling of all datasets (normal/benign findings vs. malignant lesion)	Train–validation (80–20% with a 5-fold cross-validation)	Normal/benign vs. malignant BSs	94.7%	92.1%	0.988
Pereira et al., 2022 [45]	Develop and validate a CNN-based model for automatic detection of tumor vessels using DSOC images	1	85	6475	4415	Xception	Frame labeling of all datasets (presence or absence of TV)	Train–validation split (80–20%)	Benign finding or TV	99.6%	99.4%	1
Marya et al., 2023 [37]	Develop a CNN model capable of accurate stricture classification and real-time evaluation based solely on DSOC images	2	NK	2,388,439	NK	ResNet50V2 (Exper t-CNN)	Annotation by expert (High-Quality Benign; High-Quality Malignant; High-Quality Suspicious; Low-Quality; Uninformative)	Train–validation split (80–20%)	High-Quality Benign; High-Quality Malignant; High-Quality Suspicious; Low-Quality; Uninformative	93.3%	88.2%	0.941
Robles-Medranda et al., 2023 [46]	Develop a CNN model for detecting neoplastic lesions during real-time DSOC	5	NK	CNN1: 818,080; CNN2: 198,941	NK	YOLO	Frame labeling of all datasets (neoplastic vs. non-neoplastic)	Train–validation (90–10%)	Neoplastic vs. non-neoplastic	90.5%	68.2%	NK
Zhang et al., 2023 [38]	Develop MBSDeiT, a system aiming to (1) identify qualified DSOC images and (2) identify malignant BSs in real time	3	NK	NK	NK	DeiT (Data-efficient Image Transformer)	Annotation Model 1: Qualified/Unqualified Model 2: Cancer/Non-cancer	Train, validation, internal and external testing, prospective testing and video testing	Model 1: Qualified/ Unqualified Model 2: Cancer/Non-cancer	95.6% (identifying malignant BSs with quality control AI model	89.1% (identifying malignant BSs with quality control AI model)	0.976 (identifying malignant BSs with quality control AI model)
Saraiva et al., 2023 [36]	Create a DL-based algorithm for digital cholangioscopy capable of distinguishing benign from malignant BSs	2	129	84,994	44,743	ResNet	Frame labeling of all datasets (benign vs. malignant strictures, including PP and TV)	Train–validation split (80–20%)	Normal/benign finding or malignant lesion	83.5%	82.4%	0.92
Saraiva et al., 2024 [39]	Validate a CNN model on a large dataset of DSOC images providing automatic detection of malignant BS and morphological characterization	3	164	103,082	53,678	NK	Frame labeling of all datasets (normal/benign findings or as a malignant lesion)	Train–validation split (90–10%)	Normal/benign findings; malignant lesion	93.5%	94.8%	0.96

## 3. AI as an Ally to ERCP Procedural Techniques

Regarding the therapeutic potential of ERCP, there are various potential applications of AI. Table 3 outlines the more relevant studies in this respect. Firstly, models capable of automatically detecting the ampulla and identifying the difficulty of ampullary cannulation were developed to reduce the rates of unsuccessful cannulations. It is known that the success rate for removing common bile duct (CBD) stones is around 80–85%. Therefore, the remaining 15–20% require alternative or additional techniques to achieve BD clearance. Factors associated with difficult or incomplete stone extraction include large or multiple stones, unusual stone shapes, stones located above a stricture or impacted, intrahepatic stones, altered distal BD, periampullary diverticula, and surgically modified anatomy [47]. Thus, achieving deep selective cannulation of the CBD by the proper identification of the ampulla and the correct identification of technical hard cases is crucial for procedural selection, success, and the minimization of complications.

Kim et al. explored this topic. This group developed a novel AI-assisted system using a CNN to determine the location of the ampulla and to assess the difficulty of cannulation in advance, using ERCP data from 531 and 451 patients to develop each model, respectively. This model’s performance, using a density map to identify ampulla, was comparable to human experts in recognizing the ampulla’s extent and pinpointing its location, regardless of morphological shapes, sizes, and textures. Nevertheless, the experts achieved better exclusion of irrelevant areas. Regarding the binary classification of cannulation difficulty, the model performed well in predicting easy cases, and notably, it showed a strong capability in predicting cases requiring additional techniques, with a recall of 0.564, although these cases only constituted a small portion of the data. Nevertheless, further improvements are needed to enhance the model’s clinical applicability in ERCP procedures [48].

On the other hand, AI may be beneficial for assessing, scoring, and classifying the degree of technical difficulty in the endoscopic removal of CBD stones during ERCP. In fact, in 2020, Huang et al. developed DSAS, a difficulty scoring and assistance system based on a deep CNN using CasNet for ERCP treatment of CBD stones. This group conducted a multicohort, retrospective study at three hospitals that used 1954 cholangiograms for training and testing. The study concluded that the estimation performance of the DSAS was superior to that of non-expert endoscopists and that the technical difficulty scoring performance of the DSAS was more consistent with that of expert endoscopists than two non-expert endoscopists [49]. Later, the same group validated this system through a multicenter, prospective, observational study that involved 173 additional cases. The study showed that AI accurately predicted “difficult” cases, which were associated with significantly higher rates of machine lithotripsy, longer treatment times, and increased failure rates compared to cases predicted as “easy”. Their results were consistent with expert endoscopists in the assessment of the technical difficulty scoring of CBD stone extraction during ERCP, supporting the development of standardized scores and classification systems. Also, by automatically providing a quantitative evaluation of CBD and stones, it could help endoscopists to decide on more suitable interventional techniques [50].

**Table 3:** Overview of the published work on AI in ERCP procedural techniques. AI, Artificial Intelligence; DL, Deep Learning; CNN, convolutional neural network; ERCP, endoscopic retrograde cholangiopancreatography; CBD, common bile duct; DSAS, difficulty scoring and assistance system; CAD, computer-assisted; mIoU, mean intersection-over-union; ARE, average relative error; NK, not known.

Publication Author, Year	Study Aim	Centers n	Patients N	Types of CNN	Dataset Methods	Analysis Methods	Results
Huang et al., 2020 [49]	Develop a difficulty scoring and assistance system (DSAS) for ERCP treatment of CBD stones by accurately segmenting the CBD, stones, and duodensocope	3	1560 (1954 cholangiogram images)	D-LinkNet34 and U-Net	Manual annotation by expert endoscopists of the margin of CBD, stones, and duodenoscope on the cholangiograms. After that, two expert endoscopists and two non-expert endoscopist labeled the diameter of the largest stone and of the duodenoscope, the distal CBD diameter, distal CBD angulation, and distal CBD arm	Train, internal, and external test (train: 70%–test: 30%)	**Performance of DSAS segmentation model for stones, CBD, and duodenoscope:** mIoU: 68.35%, 86.42% and 95.85%, respectively **Performance of DSAS:** ARE for stone diameter: 15.97% (95% CI: 14.04–17.90) ARE for CBD length 12.87% (95% CI: 11.18–14.57) ARE for distal CBD angulation: 5.56% (95% CI: 4.81–6.32) ARE for distal CBD arm: 15.91% (95% CI: 13.52–18.31)
Kim et al., 2021 [48]	Develop an AI-assisted ERCP procedure to accurately detect the location of ampulla of vater and to estimate cannulation difficulty	2	531 (451 for ampulla detection and 531 for cannulation difficulty)	**Ampulla identification:** U-Net (VGGNet-based encoder and decoder) **Cannulation difficulty:** VGG19 with batch normalizatio, ResNet50, and DenseNet161	**Ampulla identification:** creation of a pixel-wise soft mask (density map with the probability of whether each pixel belongs to an ampulla) **Cannulation difficulty:** frame labeling of all data sets firstly in binary classification (easy case/difficult case) and then four-class classification (easy class, class whose cannulation time was over 5 min, class requiring additional cannulation techniques, and failure class)	5-fold cross-validation	**Ampulla identification:** mIoU: 0.641, Precision: 0.762, Recall: 0.784 **Cannulation difficulty:** Easy cases Precision: 0.802, Recall: 0.719; **Difficult cases:** Precision: 0.507, Recall: 0.611
Huang et al., 2023 [50]	Develop a CAD system to assess and classify the difficulty of CBD stone removal during ERCP	3	173	CAD	Frame labeling of all datasets (difficult and easy groups)	NK	**Difficult” vs. “easy cases”** **Extraction attempts:** 7.20 vs. 4.20 (*p* < 0.001) Machine lithotripsy rate: 30.4% vs. 7.1% (*p* < 0.001) **Extraction time:** 16.59 vs. 7.69 minutes (*p* < 0.001) **Single-session clearance rate:** 73.9% vs. 94.5% (*p* < 0.001) Total clearance rate: 89.1% vs. 97.6% (*p* = 0.019)

## 4. AI for Predictive Analytics and Prognostic Models

ML holds significant value in clinical research by accurately identifying risk factors from large sets of clinical parameters and also due to its imaging analysis skills. It automates the process, reducing human errors related to data omission, multicollinearity, and overfitting in statistical analyses. This allows researchers to more precisely assess risk factors associated with specific outcomes, leading to more reliable and actionable insights [1]. Neural networks are effective at multifactorial analysis, leveraging the evaluation of biological systems, especially when it comes to prediction models. So, they are emerging as potentially useful tools for projecting clinical outcomes and can play a key role in medical decision support.

### Prognostic Models in Therapeutic ERCP

Although ERCP is a diagnostic and, most importantly, a therapeutic tool for bile duct and pancreatic conditions, it has not negligible adverse events, namely, pancreatitis, bleeding, perforation, and infections. Predictive tools to correctly select patients that will benefit from ERCP and to evaluate post-ERCP complications are necessary. Indeed, ANN models have proven to be more effective than logistic regression models at predicting the likelihood of CBD stones and thus discriminating patients who will benefit from ERCP [51]. In addition to ANN models based only on clinical data, more recently, models have also been created that integrate images (computer tomography and abdominal ultrasound) and, in this way, contribute to a more careful selection of patients for ERCP [52,53].

Post-ERCP pancreatitis (PEP) is the most common complication, occurring in about 15% of high-risk procedures and 8% of average-risk procedures [54]. Previously, studies for PEP evaluation identified single risk factors with standard statistical approaches and limited accuracy. At present, various studies demonstrate that ML models based on clinical risk factors outperform logistic regression for predicting PEP. They were also able to identify new clinical features relevant to the risk, most being pre-procedural [55,56]. A recent multicenter study developed and validated a model incorporating multimodal data through multiple steps to evaluate risk factors associated with PEP. Data were selected from 1916 ERCP procedures, and, through literature research, 49 features from electronic health records (EHRs) and 1 image feature were identified. Then, the EHR features were categorized into baseline, diagnosis, technique features, and prevention strategies, and eight models were incrementally created (1–4 incorporated feature categories and 5–8 added the image feature). Prior pancreatitis, nonsteroidal anti-inflammatory drug use, and difficult cannulation were identified as the three most relevant EHR factors. While technique features proved important, image features emerged as the most critical in enhancing the prediction of PEP [57].

The overall findings support the potential of DL technology to improve prognostic models in pancreaticobiliary therapeutic endoscopy and potentially mitigate unnecessary procedures, helping to identify the need for early intervention and enabling improvements in clinical outcomes.

## 5. Integration of AI with Other Technologies in Pancreaticobiliary Endoscopy

### 5.1. Telemedicine and Remote Consultation

AI is revolutionizing telemedicine by enhancing efficiency, accuracy, and the overall quality of healthcare delivery. One of its key applications lies in chatbots. Chatbots are computer programs designed to simulate conversations through text, image, audio, or video with human users [58]. Since their emergence, we have witnessed exponential growth, largely driven by the application of Natural Language Processing (NLP). As a result, chatbots can understand and respond appropriately to users’ requests. A significant advancement has been made with the integration of generative AI and Large Language Models (LLMs), such as ChatGPT, enabling more natural and human-like conversations and interactions.

The application of chatbots in healthcare is a relatively recent topic, where failure can result in significant concerns. The one-size-fits-all approach of LLMs is not suitable in this domain, which requires a more personalized approach [59]. A systematic review of the benefits of ChatGPT’s applications in the medical field highlighted its ability to streamline tasks, support clinical decision-making, enhance communication, and optimize patient care delivery [60]. Within the same framework, Laymouna et al. developed a rapid review with the aim of providing an in-depth analysis of the functional roles of chatbots, evaluating the specific demographics they serve, and closely examining their potential and stated advantages, as well as the limitations of these cutting-edge medical tools. Their review included 161 studies and concluded that the roles of chatbots are primarily divided into two themes: first, the delivery of remote health services, including patient support, care management, education, skills building, and health behavior promotion, and second, the provision of administrative assistance to health care providers, which includes health-related administrative tasks and research purposes. The benefits of chatbots were also categorized into two themes: first, the improvement in health care quality, encompassing improvement in health outcomes and patient management, promotion of patient-centered care, and health equity, and second, efficiency and cost-effectiveness in health care delivery. The identified limitations included ethical, medico-legal, and security issues, technical challenges, user experience problems, and socioeconomic impacts [58].

Regarding the role of chatbots in gastroenterology, we envision their potential applicability in pre-procedural guidance, offering patients tailored information to prepare for procedures; tele-radiology and tele-endoscopy, facilitating remote consultations and diagnostics; and also post-procedure monitoring, tracking recovery, and managing complications remotely, reducing the need for unnecessary in-person visits, especially critical in areas with limited access to specialized care. Finally, in documentation support, automating the conversion of spoken observations into structured medical reports saves time and effort for practitioners during procedural reporting.

Despite their promise, AI-powered chatbots face limitations, including concerns about ethical issues, biases, and accuracy. Thus, these tools must be seen as complements to, rather than replacements for, human expertise.

### 5.2. Short Reflection About the Future: Multimodal Data Fusion

Multimodal data fusion comprises fusion techniques focused on integrating information from various medical data sources—such as radiomics, genomics, and electronic health records—for comprehensive analysis and decision-making, ushering in a new era of personalized medicine. Radiomics focuses on extracting quantitative data, or features, from medical images modalities, such as computer tomography (CT), magnetic resonance imaging (MRI), and position emission tomography (PET) scans, enabling the finding of potential imaging biomarkers and hidden patterns. AI has the potential to revolutionize this radiologic area by identifying clinically relevant image biomarkers, automating workflows, and increasing diagnostic accuracy [61]. On the other hand, genomics encompasses DNA sequencing, gene expression profiles, and other molecular characteristics. At present, sequencing costs are no longer the main barrier, and the challenge lies in analyzing vast genomic data. AI and DL now enable precise variant detection, structural variation analysis, and pharmacogenomics. The lower costs and AI-driven analytics could potentially allow whole-genome sequencing (WGS) to be routinely used in clinical decisions, tailoring treatments to individual genetic profiles [62]. Additionally, the Artificial Intelligence, Radiomics, Oncopathomics, and Surgomics (AiRGOS) project suggests that the fusion of WGS, radiomics, and pathomics enhances precision medicine and can improve surgical decision-making and patient outcomes in a cost-effective way [63]. Building on this approach, the integration of multimodal AI techniques extends beyond genomics and radiomics, offering a more comprehensive view of patient data. By incorporating diverse data sources, such as imaging, pathology, and clinical records, multimodal AI enhances decision-making in precision medicine, particularly in fields like oncology and neurology [64]. Preliminary studies on multimodal AI model data fusion for precision oncology have been developed in the gastroenterology field, outperforming single modality models. Weit et al. showed that their hybrid model, by integrating radiomics and Deep Learning features from both PET and CT images, enhances diagnostic accuracy and model robustness in distinguishing PDAC and AIP [65]. Also, Cui et al. showed that the integration of EUS images and clinical data outperformed single-modality models used to diagnose solid pancreatic lesions. Notably, the model demonstrated strong performance across diverse populations, underscoring its broader applicability [66].

Another promising initiative, the IMAGene project, seeks to develop a cancer risk prediction algorithm by integrating clinical, radiomic, DNA methylation biomarkers, and environmental data to detect pancreatic cancer early in high-risk, asymptomatic individuals [67].

The future of AI lies in multimodal data fusion, combining imaging, molecular, and genomic data to create comprehensive disease profiles. By generating holistic patient-specific profiles, this approach enables personalized prevention, diagnosis, and treatment strategies—ultimately advancing precision medicine and improving patient outcomes.

## 6. Ethical and Regulatory Considerations

The integration of AI into clinical practice introduces numerous bioethical challenges that must be addressed before implementing any model. These include concerns about privacy, data protection, biases in training data, the explainability of AI tools, accountability for outcomes, patient trust in clinicians, and the adaptability of AI systems [68]. To ensure proper compliance, the FAIR principles—findable, accessible, interoperable, and reusable—have been established as guiding standards for responsible AI use [69].

One of the most pressing issues is data privacy and security. Digital data are highly vulnerable to replication, remote access, and manipulation, with potentially profound and lasting personal consequences for patients. While patient de-identification was initially proposed as a solution, it soon became clear that re-identification is alarmingly easy. Blockchain technology has since emerged as a promising alternative [68]. By storing data in cryptographically linked, decentralized blocks, blockchain ensures tamper-proof and transparent record-keeping without relying on a central authority. In healthcare, blockchain can facilitate secure data sharing and validation, enhancing trust while safeguarding privacy and ensuring compliance with regulatory standards [70].

AI systems must also align with healthcare regulations to protect patient confidentiality. Initiatives have already begun to address the legal implications of AI, particularly in areas such as digestive healthcare. Frameworks like the EU and UK’s General Data Protection Regulation (GDPR) and the USA’s Health Insurance Portability and Accountability Act (HIPAA) play a crucial role in maintaining data confidentiality and compliance.

Another critical challenge lies in addressing biases within AI models. These biases often stem from incomplete, non-representative, or misinterpreted training data, which can limit the real-world applicability of AI tools. For example, datasets that fail to adequately represent certain populations—such as variations by ethnicity or socioeconomic status—may produce inequitable outcomes [71]. Expanding datasets and integrating blockchain-enabled data from diverse healthcare platforms could help mitigate this issue by ensuring better representation and improving model reliability.

The “black-box” phenomenon is another significant concern. Many AI systems operate as opaque tools, offering little to no insight into how their conclusions are reached. These systems are often evaluated only in terms of inputs and outputs, without transparency into the underlying algorithms. While such systems can outperform physicians in detecting certain conditions, the ultimate responsibility for interpreting AI diagnoses still rests with clinicians [68]. This underscores the need for explainability and interpretability in AI systems to enhance trust and usability.

To address these concerns, Software as a Medical Device (SaMD) has gained prominence, particularly in digestive healthcare. SaMD assists in detecting clinically relevant lesions while maintaining the physician’s ultimate responsibility for patient care. Moreover, these tools are governed by a robust regulatory framework, with oversight from organizations like the International Medical Device Regulators Forum (IMDR), to ensure their safety and effectiveness [72].

Transparency throughout the AI development and implementation process is essential, particularly regarding data sources and system design. Ensuring informed consent from participants is equally critical to maintaining ethical standards. By addressing these multifaceted challenges, AI can be responsibly integrated into healthcare, paving the way for innovation while safeguarding patient rights and trust.

## 7. Future Challenges

Although the future appears highly promising regarding the implementation of AI in the medical field, we can already foresee some challenges. Firstly, limited studies had accurate external validation, leading to a small number of high-evidence studies. Indeed, compared to plain endoscopy, AI development for EUS and ERCP remains less advanced. This disparity can be explained by the difference in the availability of high-quality annotated data. Addressing this limitation will require the establishment of a worldwide system to collect and utilize EUS and ERCP images.

Future AI systems must incorporate real-time feedback mechanisms and enhance cross-platform compatibility. Although several study models with these features already exist, multicenter trials are needed to validate them across diverse clinical settings. A significant gap also lies in the current regulatory landscape. While existing frameworks could be adapted to regulate AI in clinical practice, the ideal solution would involve creating new regulatory frameworks and guidelines.

Additional barriers to the adoption of this new tool include the lack of clinician training and the hesitancy to rely on AI. Developing intuitive interfaces and providing educational resources can facilitate its smooth integration into clinical workflows.

## 8. Conclusions

AI is revolutionizing the field of pancreaticobiliary endoscopy, particularly in the domains of EUS and ERCP. By leveraging sophisticated algorithms and multimodal data fusion, AI has enhanced diagnostic accuracy, procedural efficiency, and real-time decision-making. In EUS, AI excels at differentiating pancreatic masses, predicting malignancy, and improving the training of endoscopists, while in ERCP, AI aids in diagnosing IDBS, optimizing procedural techniques, and predicting complications. AI has the potential to transform pancreaticobiliary healthcare, paving the way for a future of personalized medicine with more precise and effective patient care.

## Abbreviations

The following abbreviations are used in this manuscript:

AI	Artificial Intelligence
EUS	endoscopic ultrasound
ERCP	endoscopic retrograde cholangiopancreatography
ML	Machine Learning
DL	Deep Learning
ANN	artificial neural network
DSOC	digital single-operator cholangioscopy
CADe	Computer-Assisted Detection
CADx	Computer-Assisted Diagnosis
PCL	pancreatic cystic lesion
MCN	mucinous cystic lesion
IPMN	intraductal papillary mucinous neoplasm
SCN	serous cystic neoplasm
PPC	pancreatic pseudocyst
AUC	area under the curve
PDAC	pancreatic ductal adenocarcinoma
PNET	pancreatic neuroendocrine tumor
AIP	autoimmune pancreatitis
CP	chronic pancreatitis
CNN	convolutional neural network
LSTM	long short-term memory
EUS-nCLE	endoscopic ultrasound-guided needle-based confocal laser endomicroscopy
CAD	computer-aided detection and diagnosis
BD	bile duct
EUS-FNA	endoscopic ultrasound-guided fine needle aspiration
EUS-FNB	endoscopic ultrasound-guided fine needle biopsy
MOSE	macroscopic on-site evaluation
HSI	hyperspectral imaging
RGB	Red, Green, Blue
CE-EUS	contrast-enhanced endoscopic ultrasound
RCT	randomized-controlled trial
PPV	positive predictive value
NPV	negative predictive value
IDBS	indeterminate biliary stricture
CBD	common bile duct
PEP	Post-Endoscopic Retrograde Cholangiopancreatography Pancreatitis
EHR	electronic health record
NLP	Natural Language Processing
LLM	Large Language Model
CT	computer tomography
MRI	magnetic resonance imaging
PET	position emission tomography
WGS	whole-genome sequencing
GDPR	General Data Protection Regulation
HIPAA	Health Insurance Portability and Accountability Act
SaMD	Software as a Medical Device
IMDR	International Medical Device Regulators Forum
SEN	sensitivity
SPE	specificity
MLP	multilayer perceptron
CEH-EUS	contrast-enhanced harmonic endoscopic ultrasound
NP	normal pancreas
ADC	adenocarcinoma
CPP	chronic pseudotumoral pancreatitis
MFP	mass-forming pancreatitis
PCN	pancreatic cystic neoplasm
M-PCN	mucinous pancreatic cystic neoplasm
NM-PCN	non-mucinous pancreatic cystic neoplasm
ROI	region of interest
NK	not known
